# Supplementary material for: Comparison of activities of transcription factor NF-κB from two jellyfish models
Source: Comp Immunol Rep. 2025 Jun 17;9:200232. doi: 10.1016/j.cirep.2025.200232 (PMC12221839; doi:10.1016/j.cirep.2025.200232)
Supplement: Supplementary file 1 [file mmc1.docx]

# *Comparative Immunology Reports*

Supplementary Material for

**Comparison of Activities of Transcription Factor NF-κB from Two Jellyfish Models**

Leah M. Williams^a,#^, Wei Wang^a^, Alexandra V. Grigoryeva^a^, Alejandra Navarro-Rosado^a^, Jada A. Peart^a^, Angela Calderon^a^, Catherine L. Gill^a^, Susan Black^a^, Kristina M. Alsante^a^, Aidan T. Lackstrom^a^, Molecular Biology Laboratory^b^, Brandon Weissbourd^c^, Mengrui Wang^a^, Christopher J. DiRusso^a^, Lianne B. Cohen^a^, Zeba Wunderlich^a^, Brian P. Grone^c^, Thomas D. Gilmore^a, #,^ *

*^a^Department of Biology, Boston University, Boston, Massachusetts, USA 02215*

*^b^Molecular Biology Laboratory (BB522), Program in Biochemistry & Molecular Biology, Boston University, Boston, Massachusetts, USA 02215*

^c^Department of Biology and The Picower Institute for Learning and Memory, MIT, Cambridge, MA, 02139, USA

*^#^*These authors contributed equally

*Corresponding author: Dr. Thomas D. Gilmore, Biology Department, Boston University, 5 Cummington Mall, Boston, MA 02215, USA; 617-353-5444 (phone); 617-353-6340 (fax); E-mail: gilmore@bu.edu

This file contains Supplementary Tables, Supplementary Figures, and Supplementary References

| **Supplemental Table 1. Rel Homology Domain (RHD) Sequences used for phylogenetic analysis (Figure 1B of text).** | |
| --- | --- |
| **Organism NF-κB** | **Amino acid sequences** |
| **>Homo_sapiens_NF-kB2 (p100)** | **PYLVIVEQPKQRGFRFRYGCEGPSHGGLPGASSEKGRKTYPTVKICNYEGPAKIEVDLVTHSDPPRAHAHSLVGKQCSELGICAVSVGPKDMTAQFNNLGVLHVTKKNMMGTMIQKLQRQRLRSRPQGLTEAEQRELEQEAKELKKVMDLSIVRLRFSAFLRASDGSFSLPLKPVISQPIHDSKSPGASNLKISRMDKTAGSVRGGDEVYLLCDKVQKDDIEVRFYEDDENGWQAFGDFSPTDVHKQYAIVFRTPPYHKMKIERPVTVFLQLKRKRGGDVSDSKQFTYYPLVEDKEEVQRKRRK** |
| **>Homo_sapiens_NF-kB1 (p105)** | **PYLQILEQPKQRGFRFRYVCEGPSHGGLPGASSEKNKKSYPQVKICNYVGPAKVIVQLVTNGKNIHLHAHSLVGKHCEDGICTVTAGPKDMVVGFANLGILHVTKKKVFETLEARMTEACIRGYNPGLLVHPDLAYLQAEGGGDRQLGDREKELIRQAALQQTKEMDLSVVRLMFTAFLPDSTGSFTRRLEPVVSDAIYDSKAPNASNLKIVRMDRTAGCVTGGEEIYLLCDKVQKDDIQIRFYEEEENGGVWEGFGDFSPTDVHRQFAIVFKTPKYKDINITKPASVFVQLRRKSDLETSEPKPFLYYPEIKDKEEVQRKRQK** |
| **>Homo_sapiens_RelA** | **PYVEIIEQPKQRGMRFRYKCEGRSAGSIPGERSTDTTKTHPTIKINGYTGPGTVRISLVTKDPPHRPHPHELVGKDCRDGFYEAELCPDRCIHSFQNLGIQCVKKRDLEQAISQRIQTNNNPFQVPIEEQRGDYDLNAVRLCFQVTVRDPSGRPLRLPPVLSHPIFDNRAPNTAELKICRVNRNSGSCLGGDEIFLLCDKVQKEDIEVYFTGPGWEARGSFSQADVHRQVAIVFRTPPYADPSLQAPVRVSMQLRRPSDRELSEPMEFQYLPDTDDRHRIEEKRKRT** |
| **>Homo_sapiens_RelB** | **PYLVITEQPKQRGMRFRYECEGRSAGSILGESSTEASKTLPAIELRDCGGLREVEVTACLVWKDWPHRVHPHSLVGKDCTDGICRVRLRPHVSPRHSFNNLGIQCVRKKEIEAAIERKIQLGIDPYNAGSLKNHQEVDMNVVRICFQASYRDQQGQMRRMDPVLSEPVYDKKSTNTSELRICRINKESGPCTGGEELYLLCDKVQKEDISVVFSRASWEGRADFSQADVHRQIAIVFKTPPYEDLEIVEPVTVNVFLQRLTDGVCSEPLPFTYLPRDHDSYGVDKKRKR** |
| **>Homo_sapiens_cRel** | **PYIEIIEQPRQRGMRFRYKCEGRSAGSIPGEHSTDNNRTYPSIQIMNYYGKGKVRITLVTKNDPYKPHPHDLVGKDCRDGYYEAEFGQERRPLFFQNLGIRCVKKKEVKEAIITRIKAGINPFNVPEKQLNDIEDCDLNVVRLCFQVFLPDEHGNLTTALPPVVSNPIYDNRAPNTAELRICRVNKNCGSVRGGDEIFLLCDKVQKDDIEVRFVLNDWEAKGIFSQADVHRQVAIVFKTPPYCKAITEPVTVKMQLRRPSDQEVSESMDFRYLPDEKDTYGNKAKKQ** |
| **>Mus_musculus_NF-kB2 (p100)** | **PYLVIVEQPKQRGFRFRYGCEGPSHGGLPGASSEKGRKTYPTVKICNYEGPAKIEVDLVTHSDPPRAHAHSLVGKQCSELGVCAVSVGPKDMTAQFNNLGVLHVTKKNMMEIMIQKLQRQRLRSKPQGLTEAERRELEQEAKELKKVMDLSIVRLRFSAFLRASDGSFSLPLKPVISQPIHDSKSPGASNLKISRMDKTAGSVRGGDEVYLLCDKVQKDDIEVRFYEDDENGWQAFGDFSPTDVHKQYAIVFRTPPYHKMKIERPVTVFLQLKRKRGGDVSDSKQFTYYPLVEDKEEVQRKRRK** |
| **>Mus_musculus_NF-kB1 (p105)** | **PYLQILEQPKQRGFRFRYVCEGPSHGGLPGASSEKNKKSYPQVKICNYVGPAKVIVQLVTNGKNIHLHAHSLVGKHCEDGVCTVTAGPKDMVVGFANLGILHVTKKKVFETLEARMTEACIRGYNPGLLVHSDLAYLQAEGGGDRQLTDREKEIIRQAAVQQTKEMDLSVVRLMFTAFLPDSTGSFTRRLEPVVSDAIYDSKAPNASNLKIVRMDRTAGCVTGGEEIYLLCDKVQKDDIQIRFYEEEENGGVWEGFGDFSPTDVHRQFAIVFKTPKYKDVNITKPASVFVQLRRKSDLETSEPKPFLYYPEIKDKEEVQRKRQ** |
| **C>Mus_musculus_RelA** | **PYVEIIEQPKQRGMRFRYKCEGRSAGSIPGERSTDTTKTHPTIKINGYTGPGTVRISLVTKDPPHRPHPHELVGKDCRDGYYEADLCPDRSIHSFQNLGIQCVKKRDLEQAISQRIQTNNNPFHVPIEEQRGDYDLNAVRLCFQVTVRDPAGRPLLLTPVLSHPIFDNRAPNTAELKICRVNRNSGSCLGGDEIFLLCDKVQKEDIEVYFTGPGWEARGSFSQADVHRQVAIVFRTPPYADPSLQAPVRVSMQLRRPSDRELSEPMEFQYLPDTDDRHRIEEKRKRT** |
| **>Mus_musculus_RelB** | **PYLVITEQPKQRGMRFRYECEGRSAGSILGESSTEASKTLPAIELRDCGGLREVEVTACLVWKDWPHRVHPHSLVGKDCTDGVCRVRLRPHVSPRHSFNNLGIQCVRKKEIEAAIERKIQLGIDPYNAGSLKNHQEVDMNVVRICFQASYRDQQGHLHRMDPILSEPVYDKKSTNTSELRICRINKESGPCTGGEELYLLCDKVQKEDISVVFSTASWEGRADFSQADVHRQIAIVFKTPPYEDLEISEPVTVNVFLQRLTDGVCSEPLPFTYLPRDHDSYGVDKKRKK** |
| **>Mus_musculus_cRel** | **PYVEIIEQPRQRGMRFRYKCEGRSAGSIPGERSTDNNRTYPSVQIMNYYGKGKIRITLVTKNDPYKPHPHDLVGKDCRDGYYEAEFGPERRPLFFQNLGIRCVKKKEVKGAIILRISAGINPFNVGEQQLLDIEDCDLNVVRCVFMFFLPDEDGNFTTALPPIVSNPIYDNRAPNTAELRICRVNKNCGSVRGGDEIFLLCDKVQKDDIEVRFVLNDWEARGVFSQADVHRQVAIVFKTPPYCKAILEPVTVKMQLRRPSDQEVSESMDFRYLPDEKDAYGNKSKKQK** |
| **>Gallus_gallus_NF-kB2 (p100)** | **PYLVIIEQPKQRGFRFRYGCEGPSHGGLPGASSEKGHKTYPTVKICNYEGMARIEVDLVTHSDPPRVHAHSLVGKQCNEAGNCVAIVGPKDMTAQFSNLGVLHVTKKNMMEIMKEKLKKQKTRNTNGLLTEAELREIELEAKELKKVMDLSIVRLRFTAYLRDSSGNFTLALQPVISDPIHDSKSPGASNLKISRMDKTAGSVRGGDEVYLLCDKVQKDDIEVRFYEDDENGWQAFGDFSPTDVHKQYAIVFRTPPYHKPKIDRPVTVFLQLKRKKG** |
| **>Gallus_gallus_NF-kB1 (p105)** | **PYLQIIEQPKQRGFRFRYVCEGPSHGGLPGASSEKNKKSYPQVKICNYVGPAKVIVQLVTNGKYVHLHAHSLVGKFCEDGVCTVNAGPKDMVVGFANLGILHVTKKKVFETLETRMIDACKKGYNPGLLVHPELGYLQAEGCGDRQLTEREREIIRQAAVQQTKEMDLSVVRLMFTAFLPDSNGGFTRRLDPVISDAIYDSKAPNASNLKIVRMDRTAGCVTGGEEIYLLCDKVQKDDIQIRFYEEDENGGMWEGFGDFSPTDVHRQFAIVFKTPKYRDVNITKPASVFVQLRRKSDLETSEPKPFLYYPEIKDKEEVQRKRQKL** |
| **>Gallus_gallus_RelA** | **PFVEILEQPKQRGMRFRYKCEGRSAGSIPGEHSTDSARTHPTIRVNHYRGPGRVRVSLVTKDPPHGPHPHELVGRHCQHGYYEAELSPERCVHSFQNLGIQCVKKRELEAAVAERIRTNNNPFNVPMEERGAEYDLSAVRLCFQVWVNGPGGLCPLPPVLSQPIYDNRAPSTAELRILPGDRNSGSCQGGDEIFLLCDKVQKEDIEVRFWAEGWEAKGSFAAADVHRQVAIVFRTPPFRERSLRHPVTVRMELQRPSDRQRSPPLDFRYLPHQGDLQCIEEKRKRT** |
| **>Gallus_gallus_RelB** | **PRLIITEQPKKTGMRFRYECEGRSAGSILGESSTEASKTLPAIELLNCQAIPEVQVTACLVWKDWPHRVHPHGLVGKDCSNGLCQVRLQPHANPRHSFSNLGIQCVKKKEIEAAIEKKLQLGIDPFKAGSLKNHQEVDMNVVRICFQASYRDGSGRTRQLSPVLSEPIFDKKSTNTSELRICRMNKESGPCTGGEELYLLCDKVQKEDIAVVFRKEPWEARADFSQADVHRQGAIVLRTPPYRCVQLSEPVQVEVFLQRLTDRARSRGCPYTYLPRERDAYGVKVKRKRGMPDLLEELSGADPYGIEAKRRKP** |
| **>Gallus_gallus_cRel** | **PYIEIFEQPRQRGMRFRYKCEGRSAGSIPGEHSTDNNKTFPSIQILNYFGKVKIRTTLVTKNEPYKPHPHDLVGKDCRDGYYEAEFGPERRVLSFQNLGIQCVKKKDLKESISLRISKKINPFNVPEEQLHNIDEYDLNVVRLCFQAFLPDEHGNYTLALPPLISNPIYDNRAPNTAELRICRVNKNCGSVKGGDEIFILCDKVQKDDIEVRFVLDNWEAKGSFSQADVHRQVAIVFRTPPFLRDITEPITVKMQLRRPSDQEVSEPMDFRYLPDEKDPYGNKAKRQR** |
| **>Capsaspora_owczarzaki** | **DLLMVTEEPAQFARFRYMSEQRERSLAGENSFPTLMVNPKYARVVPEMALVTAVLVTKMPDPHTGRQQKHWHHLGGIPAAPLEGPQRIARFDNIAVIMDKANNKDKDKSKAPVRSKDDQRCVRIMFELVFVSGNTQFYGRAISQPIYNAKLAITKISHSSGPVTGGNEVIMLCSKIRKGVTGVRMTDPTQWSVQAPSGSAWELNPQTLKADCNVPGANLFFHHQYAVVLTLPPYHTQTITAPVTVRISILDTDDETESQYVEYTYLPAEAAVRNAELAARKRRR** |
| **>Amphimedon_queenslandica** | **LEIVEQPKSRGFRFRYDCEGQSHGGLPGENSEKNRRQKTYPTVHLKGYRGRARVMVSLVTDSDPAMPHAHSIVGKNAIDGRCVVEIGPETDMYAQFTSLGILHVTKKKVPEVLTRRLLQQTTPRGQMVDQMEVVDVDMTTAQLTSEEQDEIHQQAQTLAKSMNLSVVRLCFQAFLPDENGRYTIPIDPVFSNKVYDSKAPSAGTLKICRLDRTSGSVKGGDDVFLLCDKVQKNDIEVVFYEDKQETTGGMQLQPWMAKGRFGPNDVHHQYAIVFQTPTFYNQAIEHPVQVWIALKRPSDHETSEPKPFLYLPQEFDEERIGQKRRKK** |
| **>Nematostella_vectensis** | **PYLEILEQPKPRGFRFRYPSEGPSHGGLPGQFSTSKSKSYPSVQVNNYQGPCRIVVTLVTKDEPYMLHAHSLTGKNANEEGVVTVQVGPDQHMTASFPNLGIQHVTKKNVVKVLMDRFIKWQTLQNATFAKLSEGIKDGVDLSLFGVNTAINSNKLGFDKNVALSVANQEAAKSREYAKQQAAAMDLSAVRLCFQAYLPDQDGNFTRPLKPVYSDAVLDSKEPSASQLKICRMDKNSGCVTGGDEIYLLCDKVQKDDIEIHFYEMDDITGKYTWEDLGKFSPCDVHRQFAIVFKTPPYWNIAIERPANVLVELRRKKK** |
| **>Exaiptasia_pallida** | **PYLEILEQPKSRGFRFRYPCEGPSHGGLPGEFSDSKNKSYPSVQVCNYQGPCRIVVSLVTEDEPHMPHAHSLTGKHANNDGIVTVQIGTEQGMTASFPNLGIQHVTKKKVAKTLTERYTKMQALQNATLTALATNNSTPSSFMNFGSVAREQVMASQGPFDRNLAAAVAGEETKKILKLVQEQSKTMNLSAVRLCFQAYLPDENGNFTKPLKPCISNPVYDSKAPASCQLKICRMDKNSGCVTGGDEIYLLCDRVQKDDIEIRFYENNDDGKPIWEDTGKFAPADVHRQFAIVFKTPAYHNIAIERPVEVLLELRRKSDKETSEPFTFTYSPQMFDTEQIGAKRRK** |
| **>Orbicella_faveolata** | **PYMEILEQPKQRGFRFRYPCEGPSHGGLPGQYSEKGKKSYPSVQLCNYHGPARIVVSLVTVDEPPMPHAHSLIGKNSNNGAVTVQIGPEHGMTASFPNLGIQHVTKKSVGKVLMERYIKMQTLHTATLHALTADSKGFDMELVGDQALADGETATFNRTMAEAVAAEESQKVRQMVEDQKQSMNLNAVRLCFQAYLPDDGGCFTKALPPCISNPVYDSKAPSASNLKICRMDRNSGCVTGGDEVYLLCDKVQKEDIDVMFYEIDVETGKKTWEAGGVFAPTDVHRQVAIVFKTPAYWNIATERPVKVHLELRRKSDQETSEPVEFTYQPQLFDKEQIGAKRRKK** |
| **>Aurelia_aurita** | **PYLEILLQPKQRGYRFRYNSEGITHGGIPAESTEKGNKKFPTVHIANYKGKAAICITLVTAEDPPQVHAHNLVGKDVTKGMLYCEDDKQEWVKSFTNLGIQHVTKKDLVKVLHEKLCQSYQLTQFSAAIKTEDETQSFDISALIGSFGGTDGAGTMIDESVAMAVAEEENKRLRKEAEEMAKTIDLSAVRLCIVAYLPDEAGLLTRALPAVFSKPIFDQKAAHSGQLKICRISKPSSSVNGGEEVFLLCDKVQKDDISVRFYQEDGAGNATWEGFGKFTQADVHKQSAIVFTTPPYVDKAIQRPVEVWLQLKRGKDKECSEPVKFTYRPEEYDRYKIGEKRRKG** |
| **> Clytia_hemisphaerica** | **PYPFKGAARLEITRQPKKRGYRFRYLSEGKTHGMLPGEPSDSGEKVFPSVRIVNHLGNAKVVMYLVTEEDPQFIHPHTLLIDKIPVGGYHIFDVNEDFDVQLKNVAIQHVNKQDLPTKMLDRHLQSKYIKEVYSMNQYGANPKIDIDTFASNLADKAKKQPFFDHRTKQALTGEEEIQLSLAMRKKQKEINMSSCRLCFIAFLQDPETGKFDKILQPVHSDLIIDGKKKEGAPLKIIRVSHVAGSVEGGKEVWLLSDKIDAEDTEVYFWERSQDKQEMFWEGFGEFNKTDVYKQALIVFKIPPYCNQNIDQPRTVNLQLRRKKDRNCVSDVHHFSYKPKHYDRYGLREKRRKN** |
| **>Cassiopea_xamachana** | **PYLEILRQPKQRGYRFRYSSEGNTHGGIPAVCSEKGQKTFPTVQIANYSGKAAICVSLVTVDDPPRVHAHNLVGKDTSNGLFYIEDTKKSWTHSFQNLGIQHVTKKDLITVLHNKLCQQYQLTLIPQTKPDEAFQSFDIAALVDSFGAVDGPSTIFDEPMAKLVENEKKQLYAEAEKLAKSIDLSAVRLCFQAFLPDESEKLTRSLPPVYSVPIYDQKAAHAGQLRICRISSSSSSVNGDEEVFLLCDKVQKDDIAVQFFTEEGKITWEGFGKFSHSDVHKQCAIVFRTPPYIDKAIQRPVDVFVRLRRGKDNEFSPAIPFTYKPETFDKYKIGEKRRKG** |
| **>Hydra_vulgaris** | **PYLKIERQPRKYGYRFRYKTEGVCHGGILADTDGAVNCGSSKSCPKIKVHNLNGQRAKVVLRLAAEHDNETMHIHSLVYNKTVTNGVHLLTLNEVDEVELEHVAVQQEKTKETKNFLFKERVLQSEYLKKYNSINPPIDVETFVKRLEDREDKKLINRLTGVECNNKDLQSEASSLMKDLNVHSVVLAFHCFLEDSNGRYTINLPTVYSVPIYDGKNKKGCEYKILRLSSVSGTPKGGEEVWMLCDKFDKSDVEVRFFDDTPVVPWSALAIVNKSDVHNQNLIIFRTPPYKHKVLDQPVKVKIELRAASDHMRCSKHFDYTYNNSSDDGYEVGKKRKK** |
| **>Rhopilema_esculentum** | **PYIEILKQPKQRGYRFRYISEGQTHGGITADNTNKGNKQYPSIHIGNYKGRASVCITLVTADDPVRVHPYNLVGKDVKNGMFHCDETKEQWVRSFPNLGIQNVTKKDLVRVMHEKLCQQFQLNQLSAAKDNSEITQSFDIAHLLESFGGNDEPGLAIDESVALVIAEEEKRRLLIEAEKISKEIDLSAVRLCFQAFLLDESGQITKTLPAVLSQPIYDQKAAHAGQLRICRISKTSSSVAGGEEVFLLCDKVQKEHIAVRLYQEDSAGKLTWEGFGKFSQADVHKQSAIVFRTPPYVDKAIQKPIEVKLQLKRSKDNACSEPVPFIYQPVEYDQYKIGEKRRKG** |
| **>Hydractinia_symbiolongicarpus** | **PFLVIERQPKKRGYRFRYSSEGQTHGGIPGEPDKSNDNRKVYPSLRLHGHNGQAKVVVTLVTAEDNQPHLHAHSLIVNKVMTKGFHVYEMNGESSLELKSIAIQHVVKENLLSTLMDRILQTRFVETFGWNSGLNVNGTREEMFDVSHYVQSLMDRHDDTVFYDRATAQAIAGVELENLRKKANDLAKKMQMNQCRLCFQAFLPDSHGKFTRILNPVYSDIIYDGKTKQGAMLKIIRLSKVSCSVKGDEEVWLLADKLNPDDIEVRFFEKETKLSTSMVPWEDTGKFNKTDIYKQVCLIFKTPMYHDQNIRQPANVFIQLRRKTDTSCCSEPMEFKYTPIQYDRYGIGEKRKKE** |
| **>Xenia_Sp** | **AELEILEQPKQRGFRFRYSCEGPSHGGLPGENSVKGKKTSPTVQIVNYHGKARVEVSLVTTSDPPTPHAHSLVGKNVDGACVVEVSEDTGMTAVFQNLGIQHVTKKNVKNVLKERHLKRLSLEKTSISQSSNLLLNVHTNVDSERIAEVITNEEIAKIDRIVEQEAKNMNLSSVRLCFQAYLPNENGYFETCLNPVYSNPIYDSKAAAAAELKICRVDKQSGFVQGGEEVFLLCDKVQKEDIEVRFYEEHTDDDIANIKEPWEALAKFSHTDVHRQFAIVFKTPEYWNTAIEKPVTVLMELRRKSDNERSAPFEFVYKPREFDTDQIGAKRRKT** |
| **> Pocillopora_damincornis** | **PYMEILEQPKQRGFRFRYPCEGPSHGGLPGQYSEKGKKSYPSVQLCNYQGPARIVVSLVTVDEPPMPHAHSLISKNSNNGVVTVQIGPEQGMTATFPNLGIEHVTKKMVSKVLMDRYIKMQTLHTATLNALTSGDGKVFGVAGLVDQAMVDGDRGSFDKRLAEAVAEEESQKVRAMVEEQKQSMNLNAVRLCFQAFLPDETGAFTKALPPCISNAVYDSKAPSASNLKICRMDRNSGCVKGGDEVYLLCDKVQKDDIEVIFYETEMDTGKKTWEDRGVFSPTDVHRQVAIVFKTPPYWNVAIEQPVKVQLELRRKSDQETSDPVEFTYQPQMFDNEQIGAKRRKI** |
| **>Montipora_capricornis** | **PYLEILEEPKQRGFRFRYPCEGPSHGGLPGQFSEKGKKSYPSVQLCNYQGPARIVVSLVTSDEPPMPHAHSLIGKNSNNGVVIVQIGPEHGMTASFPNLGIQHVTKKKVSQVLMERYLKMQTLHTATLNAMTADNSHFDVGALGDHATSDGDRSIFDKNLAEAIAEEEAKKVRRLVEEQENSMNLSVVKLCFQAFLPDDSGCFTKALPPCFSLPVYDSKAPSAANLKICRMDRNSGSVTGNDEVYLLCDKVQKDDIEVVFYELDPDNGKRTWENRGLFAPTDVHRQVAIVFKTPAYWNVCIERPVKVHLELRRKSDKETSESVDFTYQPQEFDKEQIGAKRRKK** |
| **>Acropora_cervicornis** | **PYLEILEQPKQRGFRFRYPCEGPSHGGLPGEYSEKGKKSYPSVQLCNYKGPARIVVSLVTCDENPMPHAHSLIGKNASNGVVTVQIGPEQGMTASFPNLGIQHVTKKNVGYVLMDRYLKMQMLHTATLNALTTDPRVFDIGAMVDEATADGDRGEFDKQIADTIAEEEASKVRSLVEKQKNSMNLSVVRLCFQAYLPDDNGCFTKALPPCFSRSVYDSKAPSAANLKICRMDRNSGCVTGSDEVYLLCDKVQKDDIAVVFYEIDENGKRSWEGLGLFAPTDVHRQVAIVFKTPAYWNIAVERPVKVHLELRRKSDDETSDPVEFTYQPQMFDKEQIGSKRRKK** |
| **>Corticium_candelabrum** | **PALDIFEQPKSRGFRFRYPCEGPSHGGLPGASSTKSRKSFPTVQLFNYEGRARIVVSLVTLDNPPRPHAHSLVGKNTRNGQCIVELGPEQKMSASFPNLGVLHETKKNVPRALLFRYAWDKFSQLERAGSLPQDLIQQAAAAVPEIDGEVVDVENRPLPPLPEEVMQPLRKQADEDAKNMNLSVVRLCFQAFLPDDSGSFTRALQPCLSDPVYDSKAPSASTLKICRLDRNSGGVNGGDEVFMLCDRVQKDDIDVKFFDKNSSLSQSASLWEANASFSPNDVHRQYAIVFKTPRYWNISITRPVEVFMQLRRRSDGEVSEPKVFTYQPAEYDAEQIGRKRKKR** |
| **>Sycon_ciliatum** | **AELTILEEPRQRGFRFRYSCEGPSHGGLPGETSEKNRKSYPTVQLRKYEGRAARVVVSLVTQDDPPRPHAHSLVGRHVVNGQCTVQIGPETNWMASFPNLGILHVTKKNVVKVLLERYALALTGPGASSVALPIMPDLKPGTAPTSSLPDEAAVIAASMEKLSEEERSQLKAMAEEASRHMNLSVVRLCFQAYLADEQGSFTKPLQPCISQPVYDSKAPSASTLKISRMDRHSGSVLGGDEVYLLCDRVQKDDIEVRFFTEGDGSDSGDGRPDGWSALGVFAPSDVHRQFAIVFKTPAYCDTSISRPVNVWIQLRRRSDNETSEAKPFTYQPQIVDTEQLARKRQKK** |
| **>Acropora_muricata** | **PYLEILEQPKQRGFRFRYPCEGPSHGGLPGEYSEKGKKSYPSVQLCNYKGPARIVVSLVTSDENPMPHAHSLIGKNASNGVVTVQIGPEQGMTASFPNLGIQHVTKKNVGLVLMDRYL KMQMLHTATLNALTIDPRVFDIGAMVDEATADGDRGEFDKQIADTIAEEEASKVRSLVEKQKNSMNLSVVRLCFQAYLPDDNGCFTKALPPCFSRSVYDSKAPSAANLKICRMDRNSGCVTGNDEVYLLCDKVQKDDIAVVFYEIDENGKRSWEGQGLFAPTDVHRQVAIVFKTPAYWNIAVERPVKVHLELRRKSDDETSDPVEFTYQPQMFDKEQIGSKRRKK** |
| **>Pocillopora_verrucosa** | **PYMEILEQPKQRGFRFRYPCEGPSHGGLPGQYSEKGKKSYPSVQLCNYQGPARIVVSLVTVDEPPMPHAHSLIGKNSTNGVVTVQIGPEQGMTATFPNLGIEHVTKKMVGKVLMDRYIKMQTLHTATLNALTSGDGKVFGVAGLVDQAMVDGDRSSFDKRLAEAVAEEESQKVRAMVEEQKQSMNLNAVRLCFQAFLPDETGAFTKALHPCISNAVYDSKAPSASNLKICRMDRNSGCVKGGDEVYLLCDKVQKDDIEVIFYETEMETGKKTWEDRGVFSPTDVHRQVAIVFKTPAYWNVAIEQPVKVQLELRRKSDQETSDPVEFTYQPQMFDKEQIGAKRRKK** |
| **>Montipora_foliosa** | **PYLEILEEPKQRGFRFRYPCEGPSHGGLPGQFSEKGKKSYPSVQLCNYQGPARIVVSLVTSDEPPMPHAHSLIGKNSNNGVVIVQIGPEHGMTASFPNLGIQHVTKKKVSQVLMERYLKMQTLHTATLNAMTADNSHFDVGALGDHATSDGDRSIFDKNLAEAIAEEEAKKVRRLVEEQENSMNLSVVKLCFQAFLPDDSGCFTKALPPCFSLPVYDSKAPSAANLKICRMDRNSGSVTGNDEVYLLCDKVQKDDIEVVFYELDPDNGKRTWENRGLFAPTDVHRQVAIVFKTPAYWNVCIERPIKVHLELRRKSDKETSESVDFTYQPQEFDKEQIGAKRRKK** |
| **>Stylophora_pistillata** | **PYMEILEQPKQRGFRFRYPCEGPSHGGLPGQYSEKGKKSYPSVQLCNYQGPARIVVSLVTVDEPPMPHAHSLIGKNSAGGVVTVQIGPEQGMTATFPNLGIEHVTKKMVGKVLMDRYIKMQTLHTATLNALTSADGKGFDVTVLGDHVDGDRSSFDKHLAEAVAEEESQKVRTMVEEQKQSMNLNAVRLCFQAFLPDESGAFTKALHPCISNPVYDSKAPSASNLKICRMDRNSGCVSGSDEVYLLCDKVQKDDIEVIFYETEMETGKKTWEDRGVFAPTDVHRQVAIVFKTPAYWNVAIEQPVKVQLELRRKSDQETSDPVEFTYQPQLFDKEQIGAKRRKK** |
| **>Dendronephthya_gigantea** | **AELEILEQPKQRGFRFRYSCEGPSHGGLPGERSAKGRKSFPSVQIINYSGKARIEVTLVTVSDPPQPHAHSLVGKNVRDGACIVEVGPDCGMTASFPNLGVQHVTKKNVLPILKERYLKKHRLEKSFNSGVSSGDQVMHVDFHDPASSGAIAEAITNEEMKSIEVIAKQEADKMNLSTVRLCFQTYLPDQDGRFVICLKPVYSHPIYDSKAAAASELKICRIDRQSGFVQGGEEIFLLCDKVQKEDIEVRFFENQEEDRPEPWEAFGKFSTSDVHRQFAIVLKTPEYWNAAIEKPVKVLLELRRKSDKETSAPVEFTYKPQEFDPEQIGAKRRKK** |

| **Supplemental Table 2. Plasmids used in this study** | |
| --- | --- |
| Mainly expression vectors for use in tissue culture and yeast cells | |
| **Plasmid Name** | **Plasmid Description** |
| pUC57-Ch-NF-κB | pUC57-Simple with Ch-NF-κB cDNA codon-optimized for expression in human cells. Synthesized by GenScript. |
| pUC57-Aa-NF-κB | pUC57-Simple with Aa-NF-κB cDNA codon-optimized for expression in human cells. Synthesized by GenScript. |
| pUC57-Ch-IκB | pUC57-Simple with Ch-IκB cDNA codon-optimized for expression in human cells. Synthesized by GenScript. |
| pUC57-Ch-BCL-3 | pUC57-Simple with Ch-BCL3 cDNA codon-optimized for expression in human cells. Synthesized by GenScript. |
| pUC57-Aa-BCL3 | pUC57-Simple with Aa-IκB cDNA codon-optimized for expression in human cells. Synthesized by GenScript. |
| pUC57-Aa-NF-κB | pUC57-Simple with Aa-NF-κB cDNA codon-optimized for expression in human cells. Synthesized by GenScript. |
| pUC57-Ch-IκB | pUC57-Simple with Ch-IκB cDNA codon-optimized for expression in human cells. Synthesized by GenScript. |
| pUC57-Ch-BCL-3 | pUC57-Simple with Ch-BCL3 cDNA codon-optimized for expression in human cells. Synthesized by GenScript. |
| pUC57-Aa-BCL3 | pUC57-Simple with Aa-IκB cDNA codon-optimized for expression in human cells. Synthesized by GenScript. |
| GBT9 | Ref. 1 |
| GB-Nv-NF-κB | Ref. 1 |
| GB-Ch-NF-κB | A linker-based BamHI-BamHI fragment encoding aa 2-477 of Co-NF-κB was subcloned into BamHI-digested pGBT9 vector. |
| GB-Aa-NF-κB | A linker-based BamHI-BamHI fragment encoding aa 2-435 of Aa-NF-κB was subcloned into BamHI-digested pGBT9 vector |
| GB-Ch-BCL-3 | A linker-based BamHI-BamHI fragment encoding aa 2-739 of Ch-BCL3 was subcloned into BamHI-digested pGBT9 vector. |
| pcDNA MYC vector | Ref. 1 |
| MYC-Ch-IκB | EcoRI-XbaI fragment encoding amino acids 2-297 of Aa-IκB was subcloned into EcoRI-XbaI digested pcDNA-MYC vector. |
| MYC-Ch-BCL3 | EcoRI-XbaI fragment encoding amino acids 2-739 of Ch-BCL3 was subcloned into EcoRI-XbaI digested pcDNA-MYC vector. |
| MYC-Aa-BCL3 | EcoRI-XbaI fragment encoding amino acids 2-651 of Aa-BCL3 was subcloned into EcoRI-XbaI digested pcDNA-MYC vector. |
| 3X-κB-luc | Ref 1 |

**Supplemental Table 3. Innate immune pathway homologs in *A. aurita* and *C._hemisphaerica***

| **Protein Name** | **Homolog Present in *C. hemisphaerica*** | **Accession Number of *C. hemisphaerica* Homolog** | **Homolog Present in *A. aurita*** | **Accession Number of *A. aurita* Homolog** |
| --- | --- | --- | --- | --- |
| TLR | + | XP_066922741.1,  TCONS_0005672  TCONS_00000159 | + | scaffold128.g8 |
| IL-1R | - | - | + | scaffold8.g29, scaffold1880.g2 |
| MYD88 | -* | - | +* | Seg116.2 |
| IRAK | -* | - | -* | - |
| TRAF | +* | XP_066932459.1  XP_066918860.1  XP_066926495.1  XP_066925993.1  XP_066918082.1  XP_066935920.1 | +* | scaffold3.g45, scaffold246.g18, scaffold347.g10, scaffold1411.g5, scaffold308.g16, scaffold308.g17, scaffold3474.g3 |
| NEMO/IKKγ | +* | XP_066910580.1 | +* | scaffold301.g3 |
| IKKα | -* | - | +* | scaffold634.g1 |
| IKKβ | +* | XP_066933320.1 | -* | - |
| TBK/IKK**ε** | +* | TCONS_00026766 | - | - |
| IκB | +* | XP_066933946.1  XP_066933186.1,  TCONS_00042973-protein | - | - |
| NF-κB | +* | XP_066928423.1, TCONS_00005923 | + | scaffold238.g7 |
| BCL-3 | + | TCONS_00046043 | + | scaffold146.g27 |
| RAC1 | +* | TCONS_00065695 | -* | - |
| PI3K | +* | TCONS_00002349, TCONS_00002351 | +* | scaffold935.g2,  scaffold2153.g5,  scaffold863.g3,  scaffold790.g12 |
| AKT | +* | TCONS_00027365 | -* | - |
| TRIF | - | - | - | - |
| TRAM | -* | - | +* | scaffold162.g6 |
| RIPK | -* | - | - | - |
| TOLLIP | - | - | + | scaffold114.g6 |
| Ubc13 | + | TCONS_00003004 | + | scaffold1623.g6 |
| ECSIT | - | - | - | - |
| UEV1A | - | - | - | - |
| TAK1 | + | TCONS_00027570,  TCONS_00027576 | + | scaffold583.g13,  scaffold16.g5 |
| TAB 1/2 | +* | TCONS_00058564 | -* | - |
| A20 | - | - | - | - |
| CTLs | +* | TCONS_0000809, TCONS_00009141 | +* | scaffold468.g11 |
| SYK | +* | TCONS_00049928 | +* | scaffold383.g12 |
| CARD9 | -* | - | -* | - |
| BCL10 | -* | - | -* | - |
| MALT1 | +* | TCONS_00022168,  TCONS_00036912, TCONS_00073355 | +* | scaffold238.g3 |
| CASP8 | +* | TCONS_00063807 | -* | - |
| CASP10 | +* | TCONS_00018475,  TCONS_00061067 | -* | - |
| RIG1 | - | - | + | scaffold111.g19 |
| MDA5 | - | - | + | scaffold494.g1 |
| MAVs | - | - | -* | - |
| TRADD | -* | - | -* | - |
| FADD | -* | - | -* | - |
| RIP1 | - | - | -* | - |
| MEKK1 | +* | TCONS_00013759 | +* | scaffold130.g23 |
| NLRs | +* | TCONS_00001838,  TCONS_00031619,  TCONS_00058987,  TCONS_00059382,  TCONS_00072663 | -* | - |
| RIP2 | -* | - | -* | - |
| TIRAP/MAL | -* | - | -* | - |

*Indicates a homolog identified by Emery et al. (2021)

**Supplemental Figure 1**

**A**

**Ch-NF-κB (477 aa)**

MNLPEVTKNPIYGSSMIGASYPMSMSVPYPFKGAARLEITRQPKKRGYRFRYLSEGKTHG

MLPGEPSDSGEKVFPSVRIVNHLGNAKVVMYLVTEEDPQFIHPHTLLIDKIPVGGYHIFD

VNEDFDVQLKNVAIQHVNKQDLPTKMLDRHLQSKYIKEVYSMNQYGANPKIDIDTFASNL

ADKAKKQPFFDHRTKQALTGEEEIQLSLAMRKKQKEINMSSCRLCFIAFLQDPETGKFDK

ILQPVHSDLIIDGKKKEGAPLKIIRVSHVAGSVEGGKEVWLLSDKIDAEDTEVYFWERSQ

DKQEMFWEGFGEFNKTDVYKQALIVFKIPPYCNQNIDQPRTVNLQLRRKKDRNCVSDVHH

FSYKPKHYDRYGLREKRRKNLPPEIYESTPPIKRQHMDYAADSPPASTASKYQPYDSAHI

NNPTLSDIVMPQGRNEYRAIVDASTRHSPAVPTTFNGPLDNYRKNHHSSKESDTDEN

**Aa-NF-κB (435 aa)**

MNIGNDIDVPEELLQQILTGNYDFNMKTDDEPYLEILLQPKQRGYRFRYNSEGITHGGIP

AESTEKGNKKFPTVHIANYKGKAAICITLVTAEDPPQVHAHNLVGKDVTKGMLYCEDDKQ

EWVKSFTNLGIQHVTKKDLVKVLHEKLCQSYQLTQFSAAIKTEDETQSFDISALIGSFGG

TDGAGTMIDESVAMAVAEEENKRLRKEAEEMAKTIDLSAVRLCIVAYLPDEAGLLTRALP

AVFSKPIFDQKAAHSGQLKICRISKPSSSVNGGEEVFLLCDKVQKDDISVRFYQEDGAGN

ATWEGFGKFTQADVHKQSAIVFTTPPYVDKAIQRPVEVWLQLKRGKDKECSEPVKFTYRP

EEYDRYKIGEKRRKGLPENLEEMLNTAKQSRPATSTISSAPADLSQFNMPEGGPLDISGI

RFFKQSNQGPFSSRK

**Ch- IκB (293 aa)**

MSNPKDKLPNQPIDSGIGDSFGPDFVPDDEKRLTEELENLNIQPSNQINTKTLLDQAFTPD

EEND**TYLH**VYIAKNSPDEAMQLIEMCPDKNLLNIQNYLGQ**TPLH**VASYVNLDKVAMSLVHH

DANLEIQDRDGKNVFHICAERGHIETLENVIKMACQTNKTNSAWNLLHSTDFEGQAPFFLA

ALNKKKQMCVTLAKLNIDVNQIDIKNGN**TPIH**EAILEQNIDYDFLEFLVKTCNLNINAQNY

AGI**TALH**LAAGRNDSNTYSKLLELGANDSFVDIRDFTPEMCGPSDSIQLLNGV

**Ch Bcl-3 (727)**

MPKPYSNHSKKAEENTERFQKALQSKMAQSVATLKTTKVEGLSNLVDAARFVEKTNSSKIT

KKAKPKPIYKGKERGKEKKSHRESKHLIVEDTDADDETSDIESEVESDSQLALLKKSVSEM

MAKIQRLESKRKRKKMPKKAHLSDYEDSSDEDVKPKKPLKKESSPPTKPINANQQLTMHSQ

QMMQMQQQYWQMQQMMNMYSNPSINPAGMMPSMPFMTTQHQQQPSFVNMNSKSASNTLRFP

KTKKPNAETRFKDSSKIEDKKSRKSVEGEAGEETDASEVYAPTAIKDPINEYLGKDPFVPT

PSFISFVDDITKDEKTVQSTVVSSTKRAAETSKVPGSDIPRALFQRNDSITNTQQISATNQ

GNPSYAGILMNLFPTGKPNPLEIRENDKRSEEINTVLRSMIGYNPVDAKPKQPKRFDLSAD

RHTHSKPLFQNLLPISKHSQSPIPSDNAPESKRFVDNATWLRECSKKMDLQDQEGD**TILH**I

LIAKEETPKAIEVIKKMQHLPSLDILNALGQ**TPLH**LAMYTKNLPVVKELLQHGSDVSLVDA

HGN**NPLH**IACEENSIEMLEIVFNEGLSHYQTATNTSAMITTLAPEYFHIINARNNQGL**AAL**

**H**IAAKEDNEVIIKFLGERGAEMNNQEGRSGN**TPLL**IALMKNNWKMASFLLERFVNVNIPNF

SNF**YPLH**FAVQGNHVEMVKALLSRGAEMGSRANDYDQNANTTEIKQLLAKEMRRRRRNRIK

LEPKSQM

**Aa-BCL3 (641 aa)**

MGDGREGQSLLQRDKEGDTFLHIATARNEHSYIEKILRTDVAKELINAQNNHGQ**TALH**IAT

IMGQGDLVKLLLQYGAILQLQNNDGD**TSIH**LATKLGKIVCLMILCENVTKEILNIGNDSGE

**VPLH**LAAKSENLQAFGLFLQKEADVKFRNEKTGM**TALH**FAVEKENKEMVEKLLELGADVNA

QATNGNTPLHSLLGAEQRNTVKLLLDKGADINAKNEAGQSPHDLADKTMQRFMTRNRKDRK

TRRPKRPKGATASDNQPEPETSTTPKPVSDPTHSNEAQLSKRPFIPLTRVQSHPGKKPRLF

EDGGASNTSQGSAAPAQRQLTSSASVDHCVQLDAWGRRLDSQSLSRGNSDEPCDDIERQEC

LKLPFTAYKSMSMPEFSNGKVDVTNSIFGQQQQTLTRFQSEGLASELSRGMNRMAFDDQSQ

GHRGAQSQEQQGAQSHGQYGALLQRQQEGEAQALSEITAYLQLDDIQTQPITTLASHDVET

CSSNVSQSYSIQELANSSVAGQNSEMSAAIALSLMPNALPPMAGGSTLPVSGASQMLLSNI

QGASLGSNAIDLDQFAASQSQTTQQPSATSQSTIIQSTSILGNHIDQALAEMGLINAQGQS

IQSLAARMPELARHVEQIANQLGLPPADILLKLIENSNRNN

**B**

**Codon Optimized Ch-NF-kB**

**ATG**AACCTGCCTGAGGTGACCAAGAACCCTATCTATGGTAGCAGCATGATCGGCGCCAGCTACCCTATGAGCATGAGCGTCCCCTATCCTTTTAAGGGCGCTGCTAGACTCGAGATCACCAGACAGCCTAAAAAAAGAGGCTACCGCTTCCGGTACCTGAGCGAGGGCAAGACACACGGCATGCTGCCCGGCGAGCCTTCTGATTCTGGCGAGAAAGTGTTCCCCAGCGTGCGGATCGTGAATCACCTGGGCAACGCCAAGGTGGTTATGTACCTGGTGACCGAGGAGGACCCCCAGTTCATCCACCCCCACACCCTGCTGATCGACAAGATCCCTGTGGGCGGCTACCACATCTTCGACGTTAATGAGGACTTCGACGTGCAGCTGAAAAACGTGGCTATCCAGCACGTGAACAAGCAGGATCTGCCTACAAAGATGCTGGATAGACACCTGCAGAGCAAGTACATCAAGGAAGTGTACAGCATGAATCAGTACGGAGCCAACCCTAAGATCGACATCGACACCTTTGCCTCCAACCTGGCCGACAAAGCCAAAAAGCAGCCTTTCTTCGACCACAGAACCAAGCAGGCACTGACCGGCGAGGAAGAAATCCAGCTGTCCCTGGCCATGAGAAAGAAGCAAAAGGAGATTAACATGAGCAGCTGTAGACTGTGCTTCATCGCCTTCCTGCAGGACCCTGAAACCGGCAAGTTCGACAAGATCCTGCAGCCGGTCCATAGCGATCTGATCATCGACGGCAAGAAGAAGGAAGGCGCCCCTCTGAAGATTATCAGAGTGTCTCACGTGGCCGGATCTGTGGAAGGAGGAAAAGAAGTGTGGCTGCTGAGCGACAAGATCGATGCCGAGGATACCGAGGTGTACTTCTGGGAACGGAGCCAGGACAAGCAGGAGATGTTCTGGGAGGGCTTTGGCGAGTTCAACAAGACAGACGTGTACAAGCAGGCCCTGATTGTGTTTAAGATCCCCCCCTACTGCAACCAGAACATCGATCAGCCCAGAACAGTGAATCTCCAACTGAGAAGAAAGAAAGACCGGAACTGCGTGTCCGATGTGCATCACTTCAGCTACAAGCCTAAGCACTACGACAGATACGGCCTGCGGGAAAAGCGGAGAAAAAACCTGCCACCAGAGATCTACGAGAGCACCCCTCCAATCAAGCGGCAACACATGGACTACGCCGCCGATTCCCCTCCTGCTTCCACAGCCTCTAAGTACCAGCCATATGATAGCGCCCACATCAACAACCCCACCCTGAGCGATATCGTGATGCCTCAGGGCAGAAATGAGTACAGAGCCATCGTCGACGCCAGCACCAGGCACTCTCCTGCCGTGCCCACAACATTCAACGGCCCTCTGGACAACTACCGGAAGAACCACCACAGCTCTAAAGAAAGCGACACCGACGAGAAC**TGA**

**Codon Optimized Aa-NF-κB**

**ATG**AATATTGGCAATGACATCGATGTGCCCGAGGAACTGCTGCAGCAGATTCTGACCGGCAACTACGATTTTAACATGAAGACCGACGACGAGCCTTACCTGGAGATCCTGCTTCAGCCAAAGCAGAGGGGCTATAGATTCCGCTATAATTCAGAGGGAATCACCCACGGCGGCATTCCTGCCGAAAGCACAGAGAAGGGAAACAAGAAGTTCCCTACAGTGCACATCGCTAACTATAAAGGGAAGGCTGCCATTTGCATTACTCTGGTGACCGCTGAGGACCCACCTCAGGTGCATGCCCACAACCTGGTGGGCAAGGACGTGACTAAGGGAATGCTGTATTGCGAGGATGACAAGCAGGAATGGGTGAAGTCTTTCACCAACCTGGGCATTCAGCACGTGACAAAGAAGGATCTGGTGAAAGTGCTGCATGAAAAGCTGTGCCAGTCTTATCAGCTGACCCAGTTCTCTGCAGCAATCAAGACTGAGGACGAGACCCAGAGCTTCGACATCAGCGCCCTGATCGGCTCCTTTGGCGGCACCGACGGCGCCGGCACCATGATCGACGAGTCCGTGGCCATGGCCGTGGCCGAGGAGGAGAACAAGAGACTCAGGAAGGAGGCTGAGGAGATGGCAAAGACCATCGACCTGAGCGCTGTGAGGCTGTGCATCGTGGCCTACCTGCCTGACGAGGCCGGGCTGCTGACACGGGCTCTGCCAGCCGTGTTCTCCAAGCCAATCTTCGACCAGAAGGCCGCCCACTCTGGTCAGCTGAAAATTTGCAGAATTTCCAAGCCAAGTAGCAGCGTGAACGGAGGAGAAGAGGTGTTCCTGCTCTGCGATAAGGTGCAGAAAGATGACATCTCCGTGAGGTTTTACCAGGAGGATGGAGCCGGCAACGCCACCTGGGAGGGCTTCGGCAAGTTTACACAGGCCGACGTGCATAAGCAGAGCGCTATTGTGTTCACCACTCCCCCATACGTGGATAAGGCCATCCAGCGCCCTGTCGAGGTGTGGCTGCAGCTCAAAAGGGGCAAAGACAAGGAATGCAGCGAACCTGTGAAGTTTACCTATCGGCCCGAGGAGTATGACAGGTATAAGATCGGCGAAAAGAGGAGGAAAGGCCTGCCAGAGAACCTGGAGGAGATGCTGAATACAGCTAAGCAGTCAAGGCCCGCCACCAGCACTATTAGCTCTGCCCCAGCCGATCTGAGCCAGTTTAACATGCCAGAGGGCGGACCTCTGGACATCTCCGGCATCCGCTTCTTCAAACAGAGCAACCAGGGACCTTTTTCTAGCCGGAAG**TGA**

**Codon Optimized Ch-IκB**

**ATG**AGCAACCCCAAGGACAAGCTGCCTAACCAGCCCATCGACTCCGGCATCGGAGATTCCTTCGGCCCCGATTTCGTGCCCGACGACGAGAAGCGGCTGACAGAGGAACTGGAAAACCTGAACATCCAGCCAAGCAACCAGATCAACACCAAGACACTGCTGGACCAGGCCTTCACCCCTGATGAGGAGAACGATACCTACCTGCACGTGTACATCGCCAAGAACAGCCCTGATGAGGCCATGCAGCTGATCGAAATGTGCCCTGACAAAAACCTGCTGAACATCCAAAACTATCTCGGCCAGACACCACTGCATGTGGCCAGCTACGTGAACTTGGATAAGGTGGCTATGTCTCTGGTGCACCACGACGCCAACCTGGAGATTCAGGACAGAGATGGAAAAAACGTGTTCCACATCTGCGCCGAGAGAGGCCACATCGAGACACTGGAAAATGTGATCAAGATGGCCTGCCAGACCAACAAGACCAATAGCGCCTGGAACCTGCTGCACAGCACAGATTTTGAAGGCCAAGCCCCTTTCTTTCTGGCCGCTCTGAACAAGAAAAAGCAGATGTGCGTGACCCTGGCTAAACTGAATATCGACGTGAACCAGATCGACATCAAGAATGGCAACACCCCTATCCACGAGGCCATCCTGGAACAGAACATTGATTACGACTTCCTGGAGTTCCTGGTTAAGACCTGTAATCTGAACATCAACGCCCAGAACTACGCCGGCATCACAGCCCTGCACCTTGCAGCTGGAAGAAACGACTCTAATACCTACAGCAAGCTGCTCGAGCTGGGCGCCAATGACTCTTTTGTCGACATCCGGGACTTCACCCCTGAGATGTGTGGCCCTAGCGACAGCATCCAGCTGCTGAACGGCGTG**TGA**

**Codon Optimized Ch-BCL3**

**ATG**CCCAAACCCTACTCCAATCACAGCAAAAAAGCCGAGGAAAACACCGAGCGGTTTCAGAAGGCCCTCCAATCTAAAATGGCCCAGTCCGTGGCCACACTGAAGACCACCAAGGTGGAAGGACTGTCCAACCTGGTCGATGCCGCTAGATTTGTTGAGAAGACCAACTCATCCAAAATCACCAAGAAAGCTAAGCCTAAGCCCATATATAAGGGCAAGGAGAGAGGCAAGGAAAAGAAGTCTCACAGAGAGTCTAAGCACCTGATCGTGGAAGATACCGACGCCGACGACGAGACTAGCGACATCGAGTCTGAGGTCGAGAGCGACTCCCAGCTGGCCCTGCTGAAGAAGAGCGTGTCCGAAATGATGGCCAAGATCCAGCGGCTGGAGAGCAAGAGAAAGCGGAAGAAGATGCCAAAAAAGGCACACCTGAGCGATTACGAGGATAGCAGCGATGAGGATGTGAAGCCTAAGAAGCCCCTGAAAAAGGAATCAAGCCCTCCTACAAAGCCCATCAACGCTAACCAGCAGCTGACCATGCACTCTCAACAGATGATGCAGATGCAGCAGCAGTACTGGCAGATGCAGCAGATGATGAACATGTACAGCAACCCTTCTATCAACCCCGCCGGCATGATGCCTAGCATGCCTTTCATGACCACACAGCACCAGCAACAACCTAGCTTCGTCAACATGAACTCTAAGAGCGCCAGCAACACCCTGCGGTTTCCTAAGACAAAGAAGCCTAACGCTGAAACCAGATTCAAGGATAGCAGCAAAATTGAGGACAAGAAGTCCAGAAAGAGCGTGGAAGGAGAGGCCGGCGAGGAAACAGACGCAAGCGAGGTGTACGCCCCTACAGCCATCAAGGACCCTATCAACGAGTACCTGGGAAAAGACCCCTTCGTGCCCACCCCTAGCTTCATCAGCTTCGTGGATGACATCACCAAGGATGAGAAGACCGTGCAGAGCACAGTGGTGTCTTCTACAAAGCGCGCCGCCGAGACAAGCAAAGTGCCTGGCAGCGACATCCCTAGAGCCCTGTTTCAGAGAAATGACTCCATCACCAACACCCAACAGATCTCTGCCACCAACCAGGGCAACCCCAGCTACGCCGGCATCCTGATGAACCTTTTTCCAACAGGCAAGCCTAACCCTCTGGAAATCAGAGAGAACGACAAGCGGTCCGAGGAAATCAACACAGTGCTGCGGAGCATGATCGGCTACAACCCTGTGGACGCCAAGCCAAAGCAGCCTAAGAGATTCGACCTGTCTGCTGATAGGCACACCCACAGCAAGCCCCTGTTCCAAAACCTGCTTCCTATCAGCAAACATAGCCAGTCTCCTATTCCTAGCGACAACGCCCCTGAGAGCAAGCGGTTCGTGGATAACGCTACATGGCTCAGAGAATGTAGCAAGAAAATGGACCTGCAGGACCAGGAGGGCGACACCATCCTGCACATCCTGATCGCCAAGGAAGAAACCCCTAAGGCCATCGAGGTGATCAAGAAGATGCAACACCTGCCCAGCCTGGACATCCTGAACGCCCTGGGCCAGACGCCCCTGCACCTGGCCATGTACACCAAGAACCTGCCCGTTGTGAAAGAGCTGCTGCAGCACGGTTCTGACGTGTCCCTGGTGGACGCCCACGGAAACAACCCTCTCCATATCGCCTGCGAGGAGAATAGCATCGAGATGCTGGAAATCGTGTTCAACGAGGGCCTGAGCCACTACCAGACCGCCACCAACACCAGCGCCATGATCACAACCCTGGCCCCGGAATACTTCCACATTATCAACGCCAGAAACAATCAGGGCCTCGCAGCCCTGCACATCGCCGCTAAAGAGGACAACGAGGTGATCATCAAGTTCCTGGGCGAACGGGGCGCCGAAATGAACAATCAGGAGGGCAGAAGCGGCAATACCCCACTGCTGATCGCCCTTATGAAAAACAACTGGAAGATGGCTTCTTTCCTGCTAGAGAGATTCGTGAACGTGAACATCCCCAATTTCAGCAATTTCTACCCTCTGCATTTCGCCGTGCAGGGCAACCACGTGGAAATGGTGAAGGCTCTGCTGAGCAGAGGAGCTGAGATGGGCAGCAGAGCCAACGACTATGACCAGAACGCCAATACCACAGAGATCAAGCAGCTGCTGGCCAAAGAAATGAGACGGAGACGGAGAAACCGGATCAAGCTGGAACCTAAGAGCCAGATG**TGA**

**Codon Optimized Aa-BCL3**

**ATG**GGCGACGGCCGCGAAGGCCAGAGCCTGCTGCAGAGGGACAAAGAGGGAGATACCTTTCTGCATATCGCCACTGCCAGGAATGAGCACAGCTACATTGAGAAAATCCTGCGGACCGATGTGGCCAAAGAGCTGATAAACGCCCAGAATAATCACGGACAGACCGCCCTGCACATCGCTACTATTATGGGGCAGGGCGACCTGGTGAAACTGCTCCTGCAGTATGGCGCCATTCTGCAGCTGCAGAACAACGATGGCGACACCTCCATTCACCTGGCCACCAAACTGGGCAAAATTGTGTGCCTGATGATCCTGTGCGAGAACGTGACAAAGGAAATCCTGAACATCGGCAACGACTCTGGGGAAGTGCCTCTGCACCTGGCTGCTAAGTCAGAGAATCTGCAGGCCTTTGGCCTGTTCCTGCAGAAAGAGGCCGATGTGAAGTTCCGCAACGAGAAAACTGGCATGACCGCTCTGCACTTTGCTGTCGAGAAGGAGAACAAGGAGATGGTTGAAAAGCTGCTGGAGCTGGGCGCCGACGTGAACGCACAGGCCACTAACGGCAACACTCCTTTGCACAGCCTGCTGGGAGCAGAGCAGCGGAATACAGTCAAGCTGCTGCTGGACAAGGGCGCCGACATCAACGCCAAGAATGAGGCCGGGCAGAGTCCCCACGATCTGGCAGATAAGACCATGCAGAGGTTTATGACCCGGAACAGAAAGGACAGAAAGACAAGGAGGCCAAAGAGACCTAAGGGTGCCACTGCTTCAGACAACCAGCCCGAGCCCGAGACATCCACAACCCCTAAGCCCGTGTCAGACCCCACACATTCTAACGAAGCACAGCTGTCCAAGAGACCCTTTATTCCCCTGACCAGGGTGCAGAGTCACCCCGGCAAGAAGCCCAGGCTGTTCGAGGACGGCGGCGCCAGCAACACCAGTCAGGGGTCTGCTGCCCCAGCTCAGAGACAGCTGACCAGCTCCGCATCCGTGGATCACTGCGTGCAGCTGGACGCATGGGGACGCCGGCTGGATAGCCAGAGCCTGAGCAGGGGCAATTCTGATGAGCCCTGCGACGACATCGAGAGGCAGGAGTGTCTGAAGCTGCCATTCACCGCCTATAAGTCCATGAGCATGCCCGAGTTCAGCAATGGGAAGGTGGACGTTACTAACAGCATCTTCGGGCAGCAGCAGCAGACCCTGACCCGGTTCCAATCTGAGGGACTGGCCTCCGAGCTGTCCAGGGGCATGAACAGGATGGCCTTTGACGACCAGTCTCAGGGCCACCGCGGCGCCCAGAGCCAGGAGCAGCAGGGCGCCCAGAGCCATGGGCAGTACGGAGCTCTGCTGCAGCGCCAGCAGGAGGGAGAAGCCCAGGCCCTGAGCGAGATCACCGCCTATCTGCAGCTGGACGATATCCAGACACAGCCTATCACAACCCTGGCCTCCCACGACGTTGAAACATGCTCTTCTAACGTCAGCCAGTCCTACAGTATCCAGGAGCTGGCCAACTCATCAGTGGCCGGGCAGAATAGCGAGATGTCCGCCGCCATCGCCCTGAGTCTGATGCCAAACGCCCTGCCACCCATGGCCGGCGGCTCCACCCTCCCAGTGTCCGGAGCCTCCCAGATGCTGCTGTCAAACATTCAGGGAGCCAGTCTGGGGAGCAACGCCATCGATCTGGACCAGTTCGCCGCCTCTCAGAGCCAGACAACCCAGCAGCCTAGCGCAACCTCCCAGTCAACCATCATCCAGAGCACAAGCATCCTGGGCAACCACATTGACCAGGCCCTCGCCGAGATGGGCCTGATCAATGCTCAGGGCCAGAGCATTCAGAGCCTGGCCGCCAGGATGCCCGAGCTGGCCCGGCATGTGGAACAGATCGCCAACCAGCTGGGCCTGCCCCCAGCCGACATCCTGCTGAAACTGATCGAAAACAGCAACCGGAATAAT**TAG**

**C**


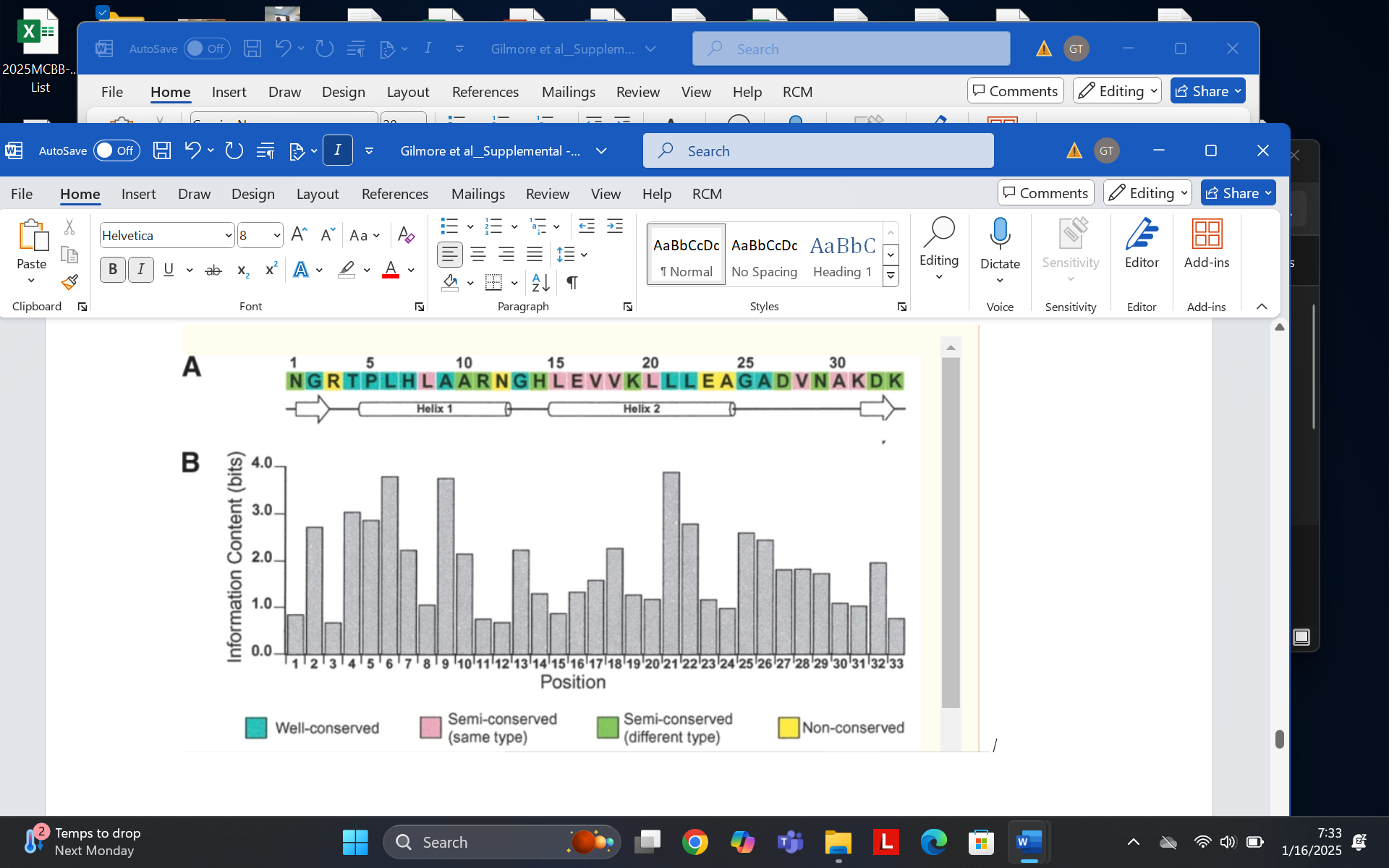


**Supplemental Fig. 1. Amino acid (A) and human cell codon-optimized nucleotide (B) sequences of the proteins used in this study.** In (A), for Ch-IκB, Ch-BCL3, and Aa-BCL3, possible ankyrin repeat sequences are underlined with conserved residues shown in green font and the core TPLH sequences in **bold green font**. In (B), the initiating ATG codons are in **bold font**, and the stop codons for each protein are in **bold underlined font**. In (C), the ANK Repeat Likely Hood Profile used to predict the ANK repeats in the sequences in (B) is shown.

**Supplemental Figure 2**

**Comparison of Aa (top) to Ch (bottom) NF-κB proteins**


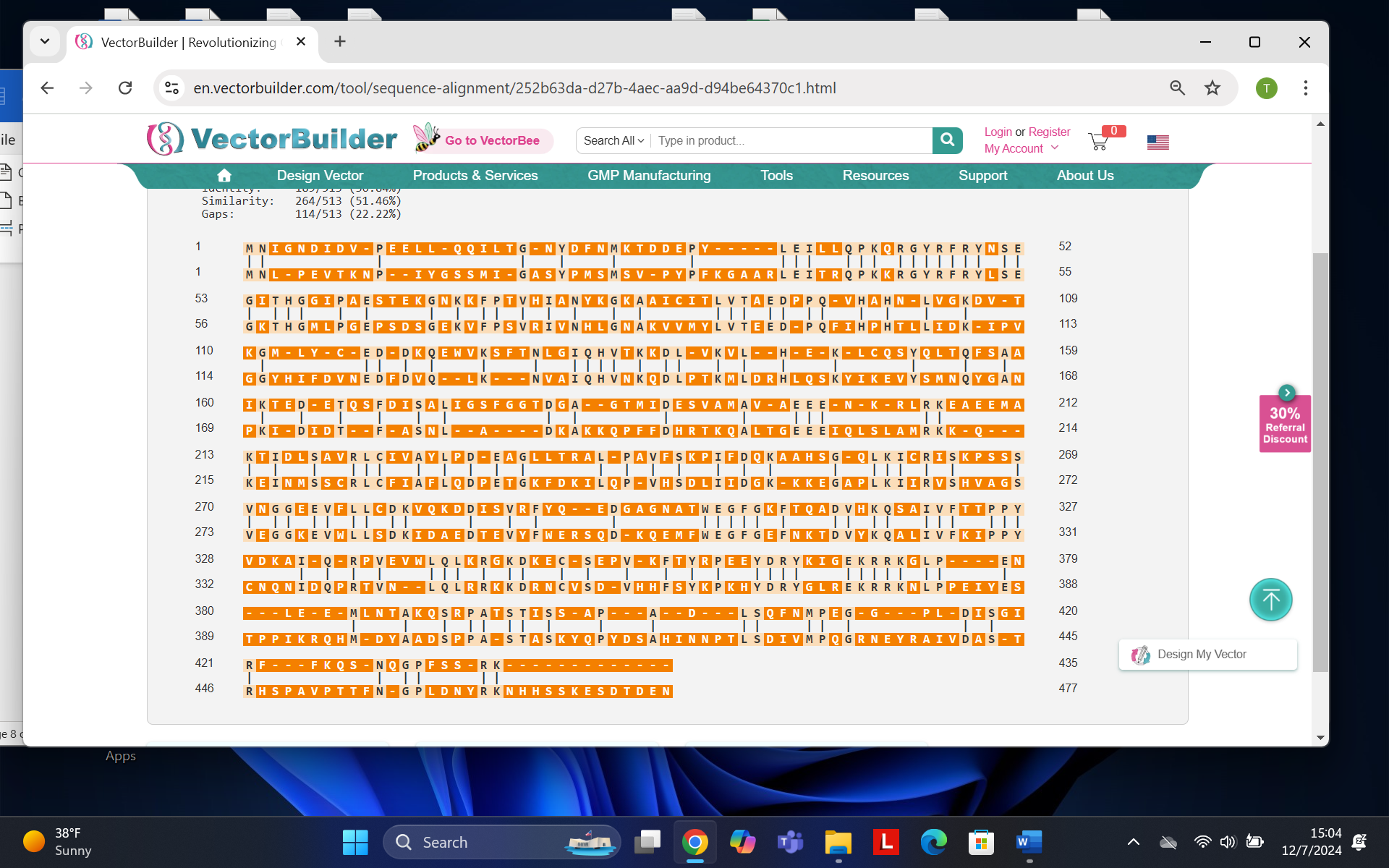


**Supplemental Fig. 2. Comparison of Aa-NF-κB (top) to Ch-NF-κB (bottom).** Comparison was done using the alignment tool at Vector Builder (<https://en.vectorbuilder.com/tool/sequence-alignment/252b63da-d27b-4aec-aa9d-d94be64370c1.html>). Note the conserved nuclear localization signal (KRRK) at aa 371-374 in Aa-NF-κB and at aa 376-379 in Ch-NF-κB.

**Supplemental Figure 3**

**
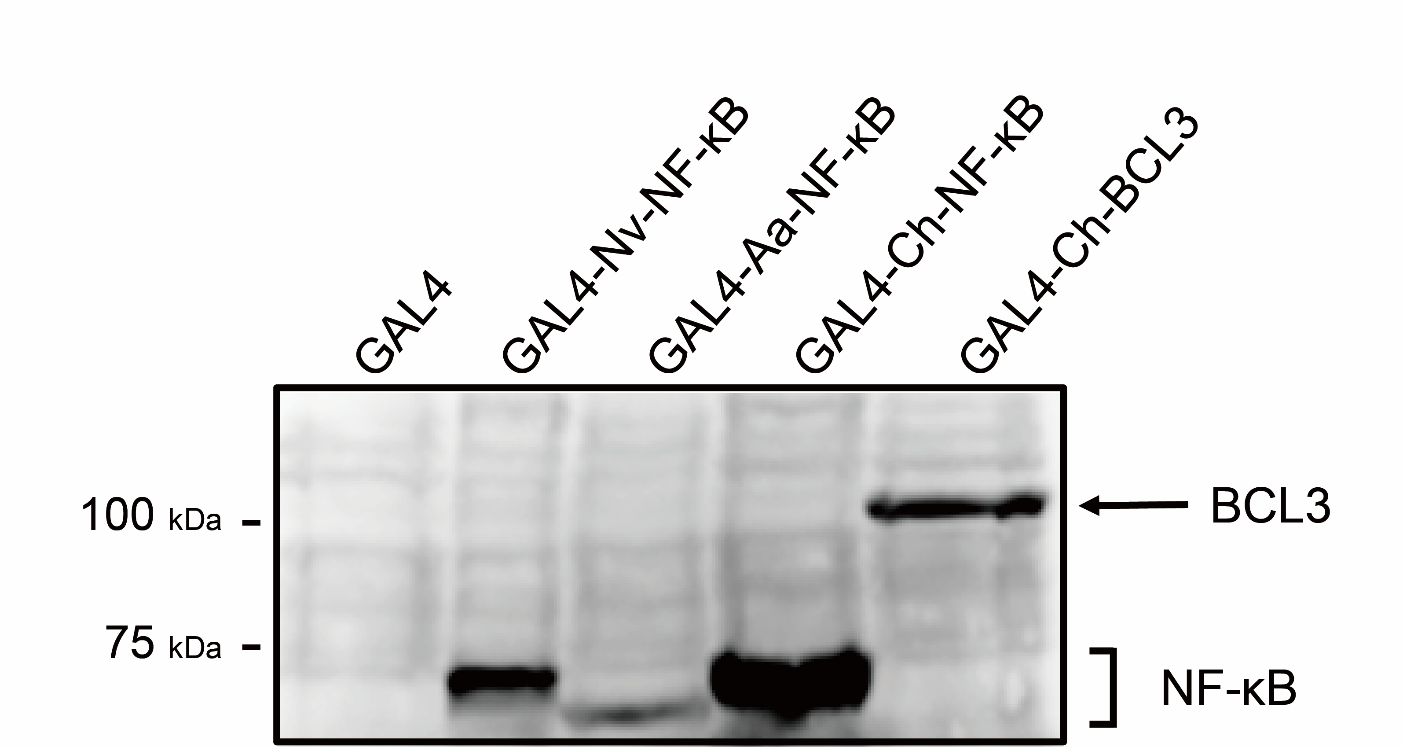
**

**Supplemental Fig. 3. Western blot of GAL4-fusion proteins used in GAL4-site reporter assays in yeast.** Shown is an anti-GAL4 Western blot of yeast cells that were transformed with expression plasmids for the indicated GAL4 proteins that were used in the reporter gene assays shown in Fig. 3F in the main text. The GAL4 (aa 1-147) vector alone protein (GAL4) is not seen in this gel because it is too small to be detected on this gel (i.e., is only approximately 17 kDa). The GAL4-NF-κB proteins all migrate at approximately 65 kDa and the GAL4-Ch-BCL3 protein is approximately 115 kDa. The migrations of the relevant proteins are indicated to the right, and the molecular weight markers are indicated to the left.

**Supplemental Figure 4**

**Ch-IκB** D**S**GIGD**S (aa 14-20)**

**Nv-IκB** D**S**GFGG**S (aa 41-47)**

**Hs-IκBα** D**S**G-LD**S (aa 31-36)**

**Supplemental Fig. 4. Comparison of possible IκB kinase (IKK) serine phosphorylation sites in Ch-IκB to other known IKK phosphorylation sites in IκB proteins.** Shown is an amino acid comparison of the indicated amino acid sequences from human (Hs), *N. vectensis* (Nv) and *C. hemisphaerica* (Ch) IκB proteins. S residues that are conserved phosphorylation sites are in **bold**. S residues in **bold red font** have been experimentally determined to be sites of phosphorylation by IKK protein kinases.

**Supplementary Reference**

1. Wolenski, F.S., Garbati, M.R., Lubinski, T.J., Traylor-Knowles, N., Dresselhaus, E., Stefanik, D.J., Goucher, H., Finnerty, J.R., Gilmore, T.D., 2011. Characterization of the core elements of the NF-κB signaling pathway of the sea anemone *Nematostella vectensis*. Mol. Cell. Biol. 31, 1076–1087.
